# Effects of Two Different Multicomponent Cognitive–Motor Training Protocols on EEG Spectral Activity, Cognitive–Motor Interference, and Gait Performance in Individuals with Chronic Stroke: A Randomized Controlled Trial

**DOI:** 10.3390/medicina62091738

**Published:** 2026-09-09

**Authors:** Shin-Young Park, Si-A Lee

**Affiliations:** Department of Physical Therapy, Korea National University of Transportation, Jeungpyeong-gun 27909, Chungcheongbuk-do, Republic of Korea; dkssud10613@naver.com

**Keywords:** stroke, task complexity, cognitive–motor interference, dual-task gait training, electroencephalography

## Abstract

*Background and Objectives*: This trial examined whether two task-complexity gait training protocols differentially affect EEG spectral activity, cognitive–motor interference, and gait performance in chronic stroke. *Materials and Methods*: Forty-four participants were randomly allocated to Progressive Task-Complexity Gait Training (PTCGT; *n* = 22) or Constant Task-Complexity Gait Training (CTCGT; *n* = 22). Both groups trained for 4 weeks using the same auditory-feedback walker. PTCGT progressed from obstacle avoidance to rhythmic auditory cueing plus obstacle avoidance, followed by a verbal fluency dual-task. CTCGT maintained rhythmic auditory cueing plus obstacle avoidance. Outcomes included EEG *theta*, *SMR*, *mid-beta*, Concentration Index, SEF-50, DTC%, and GAITRite gait parameters. *Results*: Significant group-by-time interactions were observed for EEG and gait measures. DTC showed a significant interaction in repeated-measures ANOVA (*p* = 0.041), but not in mixed-effects sensitivity analysis (*p* = 0.088). PTCGT showed greater pre–post changes in EEG and gait outcomes, whereas the DTC difference was less robust. *Conclusions*: PTCGT was associated with greater changes in EEG spectral characteristics and gait performance than CTCGT. However, these differences cannot be attributed specifically to the progressive task-complexity protocol because PTCGT also involved additional cognitive-task exposure and greater task complexity. In addition, because cognitive-task performance was not quantitatively assessed, improvements in dual-task ability or cognitive–motor integration cannot be established.

## 1. Introduction

Even when individuals with stroke are capable of independent walking, their walking performance often deteriorates significantly in complex real-life environments [1]. This is because walking in daily life is not merely a simple motor activity but a cognitive–motor integration process that requires continuous processing of information from the surrounding environment, obstacle avoidance, and appropriate adjustment of walking speed and direction [2,3].

When walking and cognitive tasks are performed simultaneously, performance may decline because limited cognitive resources must be shared between the two tasks; this phenomenon is referred to as cognitive–motor interference (CMI). This interference has been reported to be more pronounced in individuals with stroke due to reduced gait automaticity [4]. Because cognitive–motor interference is closely associated with reduced gait performance and an increased risk of falls [5], interventions aimed at simultaneously improving gait ability and cognitive control are warranted.

Dual-task gait training is widely used to address these challenges. Previous studies have reported positive effects of incorporating various cognitive tasks during walking on gait performance and cognitive function [6]. However, the degree and pattern of dual-task interference may vary according to the difficulty and configuration of the concurrent task, highlighting the importance of considering task characteristics when interpreting training effects [7]. Nevertheless, the effects of multi-component cognitive–motor gait training protocols that differ in task complexity and content have not been sufficiently investigated in individuals with chronic stroke.

In rehabilitation, task components can be introduced sequentially over the course of training, with additional components added to previously established tasks, whereas the same set of task components can be maintained throughout the intervention [8,9]. Stepwise task progression has been used in attention-focused rehabilitation to gradually increase task complexity [10,11]. This approach may also be relevant to gait training because real-world walking may require coordination among multiple task components. Maintaining the same set of task components, in contrast, may provide repeated practice under a stable task configuration [12,13]. However, randomized controlled trials comparing gait-training protocols that progressively add task components with those maintaining a consistent task configuration remain limited in individuals with chronic stroke, particularly when neurophysiological outcomes are also examined [14]. Differences in the sequence, content, and complexity of task components may influence not only behavioral performance but also EEG spectral activity [15].

Previous studies have shown that increased *theta*-band activity in the frontocentral cortical region is associated with greater cognitive demand, whereas *beta* and *SMR* band activity are linked to attentional engagement and sensorimotor integration [16,17,18].

In addition, the Concentration Index (CI), calculated as the ratio of *SMR* plus *mid-beta* power to *theta* power, has been used as an EEG-derived measure of concentration during task performance [19,20].

Therefore, examining EEG spectral changes associated with differences in task diversity and complexity during cognitive–motor gait training may provide insight into the neurophysiological responses to varying task demands. Accordingly, this study aimed to investigate differences in EEG spectral activity, cognitive–motor interference, and gait ability between two protocols in individuals with chronic stroke. Through this investigation, we sought to identify differences between the two training protocols that may inform their application according to patient characteristics and treatment objectives during cognitive–motor integrated gait training.

In this study, we hypothesized that the PTCGT protocol would produce greater pre–post changes than the CTCGT protocol in EEG spectral characteristics, DTC, and gait performance. Given the differences in task content between the protocols, this hypothesis concerns the overall effects of the two training protocols. Because the present study used a global scalp mean-based analysis rather than electrode-specific local analyses, the aim was to examine overall changes in frequency characteristics rather than localized changes within specific cortical regions.

## 2. Materials and Methods

### 2.1. Ethical Approval and Patient/Public Involvement

This study was conducted in accordance with the ethical principles of the Declaration of Helsinki. Ethical approval was obtained from the Institutional Review Board (IRB) of Korea National University of Transportation (Approval No.: KNUT-2026-HR-26-16, Approval Date: 16 June 2026).

Additionally, the study was pre-registered with the Clinical Research Information Service (CRIS) (Registration No.: KCT0012160; https://cris.nih.go.kr, Registration Date: 23 June 2026); Date of First Enrollment: 24 June 2026.

Following CRIS registration on 23 June 2026, participant recruitment was initiated, and the first participant was enrolled on 24 June 2026.

All participants voluntarily signed written informed consent forms after receiving a full explanation of the study’s purpose, procedures, and privacy protections. The study was designed and reported in accordance with the CONSORT 2025 Statement, and a participant flow diagram was included in accordance with CONSORT recommendations. Patients, caregivers, and members of the public were not involved in the design, conduct, analysis, interpretation, or reporting of this trial.

### 2.2. Subjects

This study included patients diagnosed with stroke who were receiving inpatient rehabilitation at S Nursing Hospital in Osan-si, Gyeonggi-do. Participants were recruited from among those who voluntarily provided informed consent after receiving a full explanation of the study purpose and procedures.

The inclusion and exclusion criteria were as follows:

Inclusion Criteria

- Adults aged ≥18 years who were at least 6 months post-stroke.
- Individuals with a Korean version of the Mini-Mental State Examination (MMSE-K) score of ≥24 who were able to understand study instructions.
- Individuals with a Functional Ambulation Category (FAC) score of ≥3, indicating the ability to ambulate using a walker under the supervision of a physical therapist.
- Individuals scoring ≥4 on the “standing without support” item of the Berg Balance Scale (BBS).
- Individuals with a Grip item score of ≥2 on the Manual Function Test (MFT) in the paretic upper extremity.
- Individuals with a Modified Ashworth Scale (MAS) grade of ≤1 in the paretic elbow flexors.

The MFT and MAS criteria for the paretic upper extremity were applied because adequate hand grip ability and limited elbow flexor spasticity were required for safe and consistent use of the walker during training.

Exclusion Criteria

- Individuals with a history of motor neuron diseases or psychiatric disorders.
- Individuals diagnosed with hemineglect.
- Individuals with hearing deficits that could interfere with auditory feedback.
- Individuals with apraxia that could interfere with gait training or cognitive task performance.
- Individuals with persistent dizziness.

### 2.3. Research Procedure

This study was designed as a single-blind randomized controlled trial in which outcome assessors were blinded to group allocation. A total of 61 individuals expressed interest in participating in the study and underwent eligibility screening according to the prespecified inclusion and exclusion criteria. Eligible participants completed the pre-intervention assessment and were then randomly assigned in a 1:1 ratio to either the Progressive Task-Complexity Gait Training group (PTCGT) or the Constant Task-Complexity Gait Training group (CTCGT).

Randomization was performed in a 1:1 ratio using a computer-generated randomization sequence. The sequence generation and allocation procedures were managed by an independent third party who was not involved in participant recruitment, baseline assessment, intervention delivery, or outcome assessment. Group allocation was not disclosed to the outcome assessor, who remained blinded throughout the outcome assessments. Allocation was revealed to the intervention provider only after completion of the baseline assessment. Because of the nature of the intervention, blinding of participants and intervention providers was not feasible.

Of the 61 individuals who expressed interest in participation, 17 did not meet the eligibility criteria and were excluded. The reasons for exclusion were MMSE-K score < 24 (*n* = 5), dizziness due to unstable blood pressure (*n* = 1), acute-stage stroke (*n* = 6), MAS grade of 1+ or higher in the elbow joint muscle tone assessment (*n* = 3), and inability to obtain consent from a legal representative (*n* = 2). A total of 44 eligible participants were subsequently randomized, and no participants dropped out or were excluded after randomization. All participants completed the intervention and post-intervention assessments.

**Figure 1:**
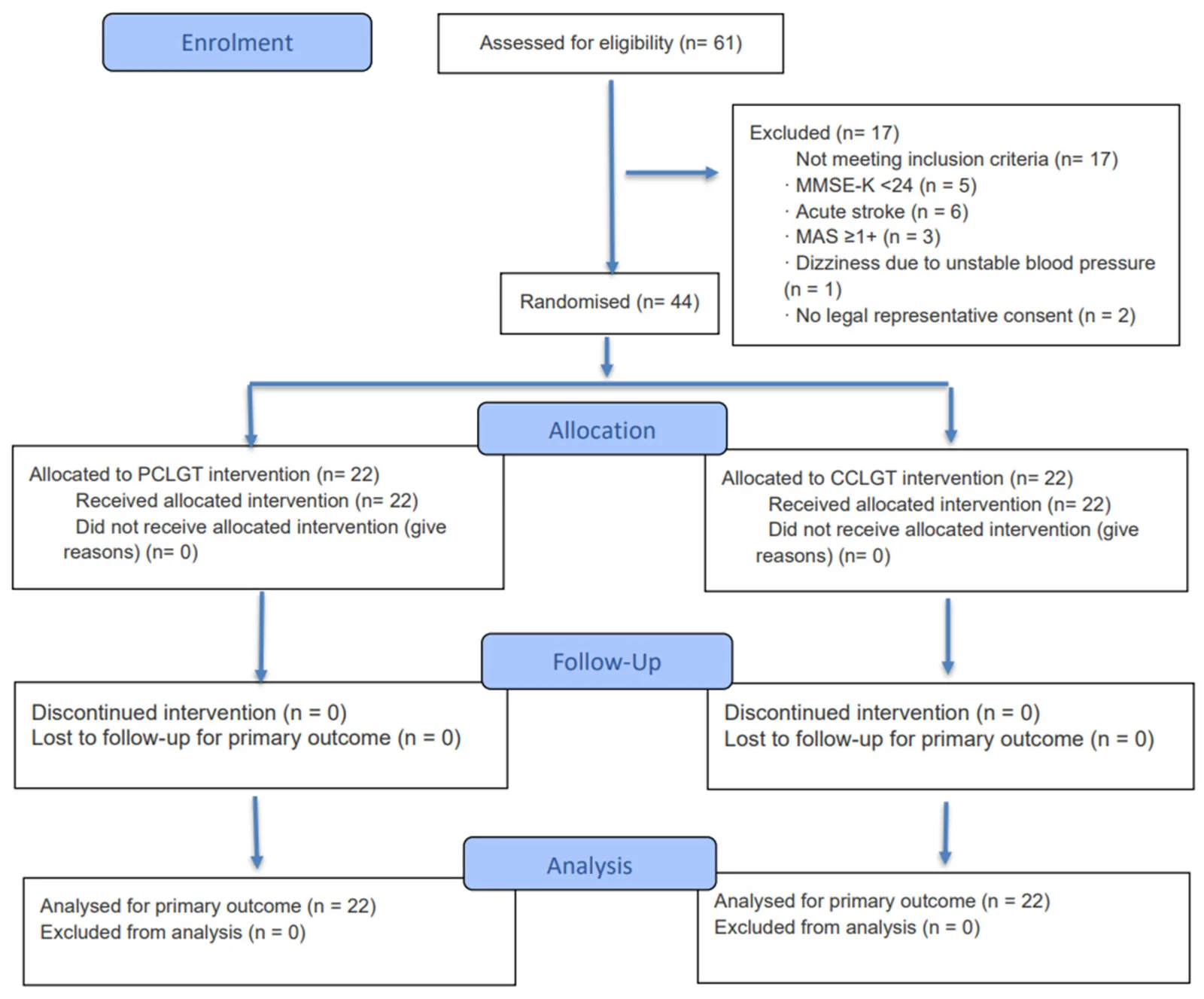
Flow diagram of the study procedure.

The study procedure flowchart, including participant selection, randomization, and intervention processes, is presented in Figure 1.

The required sample size was calculated a priori using G*Power 3.1 based on an F test for ANOVA: repeated measures, within–between interaction. The design consisted of two groups (PTCGT and CTCGT) and two measurement occasions (pre- and post-intervention). The effect size was set at Cohen’s f = 0.56, which was derived from a previously reported group × time interaction effect (partial η^2^ = 0.24) for TMT-B errors in individuals with chronic stroke [21]. The significance level (α) was set at 0.05, and the statistical power (1 − β) was set at 0.80. The analysis indicated that 19 participants per group (38 participants in total) were required. Considering an anticipated dropout rate of approximately 10%, the target sample size was set at 44 participants (22 per group).

TMT-B was selected as the reference for sample-size estimation because it is a commonly used cognitive assessment in stroke research and is conceptually relevant to the cognitive–motor and attentional processes involved in the present training protocols.

The effect size derived from TMT-B was used only as a reference for estimating the required sample size and was not used as an outcome measure or for interpreting the treatment effects in the present study.

No intervention-related adverse events were observed or reported during the training period.

### 2.4. Intervention

This study was conducted to compare the effects of gait training according to different cognitive–motor gait training protocols. Participants were divided into the Progressive Task-Complexity Gait Training group (PTCGT) and the Constant Task-Complexity Gait Training group (CTCGT).

All participants underwent training using the same sensor-based auditory feedback walker and in the same obstacle-based gait environment. Both groups used the same basic walking environment and auditory feedback system; however, the task configuration and complexity provided during training differed between the groups.

The PTCGT was designed to progressively increase task complexity by adding new task components to the preceding configuration across three sequential stages, with Stage 3 incorporating a concurrent fruit-naming verbal fluency task in addition to metronome cueing and obstacle avoidance.

The CTCGT maintained the same task configuration consisting of metronome cueing and obstacle avoidance throughout the intervention period, and no additional verbal fluency task was provided.

Both groups continued their usual inpatient rehabilitation program during the study period. Usual rehabilitation consisted of conventional physical therapy for 30 min, functional electrical stimulation (FES) for 30 min, and standing frame training for 30 min. The frequency and content of these treatments were maintained according to the existing treatment schedule of the hospital. No additional rehabilitation intervention was introduced specifically for either study group.

Intervention adherence was monitored using a tablet application connected to the sensor-based smart walker.

#### 2.4.1. Common Training Environment

**Figure 2:**
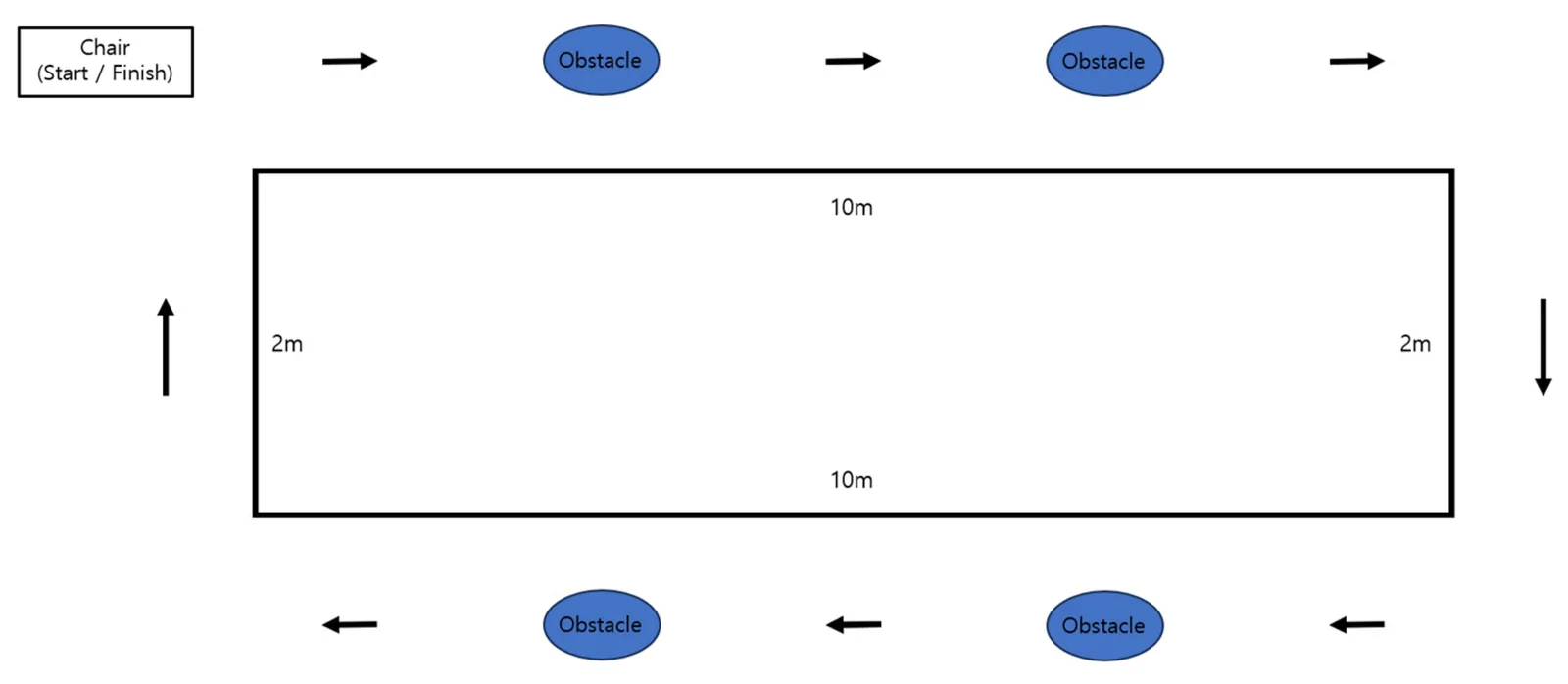
Obstacle gait training course.

Training was conducted on a 10 × 2 m walkway, and an obstacle-avoidance scenario was simulated by placing foam rollers on both sides of the walking path (Figure 2). Foam rollers measuring 90 cm in length and 15 cm in diameter were positioned at regular intervals (approximately 300 cm) along both sides of the walkway.

All participants waited while seated in chairs with armrests and stood up upon the therapist’s verbal instruction to begin gait training. They were then instructed to walk along the obstacle course using the sensor-based feedback walker. During training, the therapist provided safety supervision only and did not offer additional verbal feedback or gait correction. The supervising therapist monitored participants for falls, injuries, dizziness, pain, or other unexpected events throughout each training session.

#### 2.4.2. Rhythmic Auditory Stimulation (Metronome)

The system was configured to provide constant rhythmic auditory cues synchronized with each participant’s cadence. The metronome speed was individually determined based on the average cadence measured during a 5-min comfortable walking trial conducted prior to the intervention. Cadence was monitored in real time via a tablet connected to the sensor-based feedback walker. The metronome speed was kept constant throughout the intervention period to control for changes in training intensity. Participants were instructed to synchronize their walking speed and rhythm with the metronome cues.

#### 2.4.3. Obstacle Auditory Feedback

The system was programmed to deliver a pre-recorded voice message stating, “An obstacle has been detected ahead,” when an obstacle was detected within approximately 1 m in front of the walker. Participants were instructed to avoid the obstacle by moving to the left or right in response to this auditory cue.

The training consisted of a total of 18 sets, with one lap of the 10 × 2 m walkway defined as one set. Each set lasted approximately 1 min, followed by a 30-s rest period. The total training time per session, including both walking and rest periods, was approximately 30 min. The intervention was conducted five times per week for four weeks.

The duration of each set was determined based on the average walking time measured during the pre-intervention Dual 10-Meter Walk Test (Dual 10MWT). It was set to approximately 1 min to account for the additional demands associated with obstacle avoidance and cognitive task performance.

#### 2.4.4. Progressive Task-Complexity Gait Training Group (PTCGT)

The PTCGT was designed as a multi-component gait training protocol in which task complexity was progressively increased according to a predefined three-stage sequence. Across stages, additional task components were added to the preceding task configuration, resulting in a progressively more complex combination of task components, with reference to the stepwise principles of Attention Process Training (APT).

The training consisted of three stages, progressing sequentially from Stage 1 to Stage 3 within each session.

Stage 1: Obstacle avoidance.

Stage 2: Rhythmic auditory cueing + obstacle avoidance.

Stage 3: Rhythmic auditory cueing + obstacle avoidance + dual task.

In Stage 1, participants performed gait training while maintaining continuous attention to obstacle location and walking direction. Only obstacle-detection feedback was provided via the feedback walker. Participants were instructed to walk at a comfortable pace and to move to the left or right when an obstacle was detected.

In Stage 2, participants were required to coordinate walking, obstacle avoidance, and rhythmic auditory cueing while responding to changing environmental inputs.

This stage provided simultaneous metronome cues and obstacle-detection feedback through the feedback walker. Participants were instructed to maintain a constant walking speed, avoid obstacles by moving left or right when detected, and resume a steady walking speed synchronized with the metronome rhythm after obstacle avoidance.

In Stage 3, participants were required to perform a fruit-naming task while simultaneously performing walking and obstacle avoidance. Specifically, they were instructed to maintain the metronome rhythm, avoid obstacles, and perform the verbal fluency task concurrently.

Each stage consisted of six sets, with a 30-s rest period provided after each set.

#### 2.4.5. Constant Task-Complexity Gait Training Group (CTCGT)

The CTCGT repeatedly performed the same two-component training configuration used in Stage 2 of the PTCGT, namely metronome cueing plus obstacle avoidance, throughout the 4-week intervention period. No additional verbal fluency task was provided during training.

### 2.5. Outcome Measures

All evaluations were conducted under identical conditions before and after the intervention. The primary outcomes were predefined as attention-related neurophysiological indices (EEG-based CI and SEF-50) and EEG spectral activity (*Theta*, *SMR*, and *Mid-Beta*), whereas the secondary outcomes included cognitive–motor interference index (Dual-Task Cost, DTC%) and spatiotemporal gait-related variables.

#### 2.5.1. Attention-Related Neurophysiological Measures

##### Electroencephalography (EEG)

A mobile EEG system (Smarting mobi, mBrainTrain, Belgrade, Serbia) was used to assess neurophysiological changes during gait. EEG signals were recorded at a sampling rate of 500 Hz with 24-bit resolution and transmitted wirelessly via Bluetooth. Electrodes were placed according to the International 10–20 system at the prefrontal (Fp1, Fp2), frontal (F3, F4), sensorimotor (C3, C4), and parietal (P3, P4) regions. FCz was used as the reference electrode and Fpz as the ground electrode. Electrode impedance was maintained below 10 kΩ across all channels to minimize noise associated with skin–electrode impedance. The electrode and cable assemblies were secured to minimize movement-related artifacts during walking.

EEG data were recorded during resting-state, single-task gait, and dual-task gait conditions. Resting-state EEG was recorded for 30 s while participants were seated with their eyes open. During gait tasks, the walking conditions and procedures were standardized across participants. To reduce motion- and task-related artifacts, participants were instructed to minimize unnecessary head movements and speaking and to follow the walking path and task instructions consistently.

Raw EEG data were processed using TeleScan software (Version 3.03, Laxtha Inc., Daejeon, Republic of Korea). For gait EEG recordings, the initial and final 10 s of each recording were excluded to minimize transient artifacts associated with gait initiation and termination. The remaining data were segmented into 2-s epochs for frequency-domain analysis. Preprocessing included a 1–40 Hz band-pass filter and a 60 Hz notch filter to remove power-line interference. Independent Component Analysis (ICA) was then performed to identify and remove ocular and movement-related artifacts. ICA components were independently evaluated by two researchers based on waveform morphology, frequency characteristics, and scalp topography, and discrepancies were resolved by consensus. Epochs showing amplitude fluctuations exceeding ±100 μV were automatically excluded as residual artifacts. Data were included only when at least 20 valid epochs were available for each condition; all participants met this criterion and were therefore included in the final analysis. On average, approximately 29% of epochs were removed during artifact correction.

Artifact-corrected EEG signals were transformed from the time domain to the frequency domain using Fast Fourier Transform (FFT). Power spectral density (PSD) and Spectral Edge Frequency 50% (SEF-50) were calculated for the *Theta* (4–8 Hz), Sensorimotor Rhythm (*SMR*; 12–15 Hz), and *Mid-Beta* (15–20 Hz) frequency bands. Absolute power was calculated for each frequency band to assess intervention-related changes in band-specific EEG activity. For global scalp-level analysis, power values across all eight recording electrodes (Fp1, Fp2, F3, F4, C3, C4, P3, and P4) were averaged for each condition.

##### Concentration Index (CI)

*SMR* and *Mid-Beta* EEG activity have been reported to be associated with attentional engagement and cognitive processing, whereas *Theta* activity has been associated with cognitive demands and attentional control. Based on the relative relationship between these frequency bands, the Concentration Index (CI) has been used in previous EEG studies as an index of concentration- or attention-related states.

CI was defined as the sum of the power spectral densities of the Sensorimotor Rhythm (*SMR*) and *Mid-Beta* bands divided by the power spectral density of the *Theta* band.

- *SMR* = 12–15 Hz
- *Mid-Beta* = 15–20 Hz
- *Theta* = 4–8 Hz

The formula used was:

$$CI=\frac{SMR+Mid\;Beta}{Theta}$$

The CI formula used in the present study was identical to the definition used in previous EEG studies [22]. However, because CI is a composite index representing the relative spectral relationship among frequency bands rather than a direct measure of cognitive performance, changes in CI were not interpreted as direct evidence of improved attention or cognitive efficiency.

##### Spectral Edge Frequency 50% (SEF-50)

SEF-50 represents the frequency corresponding to 50% of the total power based on the cumulative power spectral distribution.

In the present study, SEF-50 was interpreted as a measure of changes in the distribution of scalp-recorded spectral power rather than as direct evidence of cognitive efficiency or attentional improvement.

#### 2.5.2. Cognitive–Motor Interference

##### Dual-Task Cost (DTC, %)

To quantify the degree of cognitive–motor interference under dual-task conditions, Dual-Task Cost (DTC, %) was calculated. DTC was derived using single-task and dual-task walking times obtained from the 10-Meter Walk Test (10MWT) according to the following formula:

$$DTC(\%)=\frac{Dual\;Task-Single\;Task}{Single\;Task}\times 100$$

DTC was used in the present study as an outcome reflecting the extent to which walking performance was affected under dual-task conditions.

(Higher DTC values indicate greater cognitive–motor interference under dual-task conditions, whereas lower values indicate reduced interference).

#### 2.5.3. Gait Ability

##### GAITRite System

Spatiotemporal gait variables were measured using an electronic gait analysis system (GAITRite^®^, CIR Systems Inc., Franklin, NJ, USA). To ensure steady-state walking, participants were instructed to begin walking approximately 2 m before reaching the measurement mat and to walk at a comfortable pace. Measurements were performed three times, and the average values were used for analysis.

The variables measured included gait velocity (cm/s), cadence (steps/min), step length (cm), stride length (cm), swing phase (% gait cycle), stance phase (% gait cycle), single-support phase (% gait cycle), and double-support phase (% gait cycle).

The inter-rater reliability of the GAITRite system has been reported as ICC = 0.92, and the intra-rater reliability as ICC = 0.77–0.90 depending on the variable [23].

##### 10-Meter Walk Test (10MWT)

The 10MWT was used to calculate DTC. The 10MWT was used to assess walking speed under both single-task and dual-task conditions. Because the dual-task component of the intervention involved a fruit-naming task, the category was changed to a flower-naming task during the 10MWT to minimize potential learning effects and task familiarity.

The flower-naming task is a category fluency task in which participants are instructed to name as many items as possible while walking. Walking time was recorded over the middle 10 m of a 14-m straight walkway, excluding the initial 2 m for acceleration and the final 2 m for deceleration. Time was recorded in seconds (s).

Participants were instructed to walk at a comfortable pace, and the average of three trials was used for analysis. The intra-rater reliability of the 10MWT in individuals with stroke has been reported as ICC = 0.95–0.99 [24].

### 2.6. Data Processing and Statistical Analysis

Data were analyzed using SPSS version 23.0 for Windows (SPSS Inc., Chicago, IL, USA) and R statistical software, version 4.6.1 (R Foundation for Statistical Computing, Vienna, Austria). Continuous variables were presented as mean ± standard deviation (SD), and categorical variables were presented as frequency and percentage (*n*, %). Baseline characteristics were summarized descriptively [24].

The normality of continuous outcome variables was assessed using the Shapiro–Wilk test, and homogeneity of variance between groups was assessed using Levene’s test. For variables satisfying the assumptions of parametric analysis, a two-way repeated-measures analysis of variance (ANOVA) was conducted with group (PTCGT vs. CTCGT) as the between-subject factor and time (pre- vs. post-intervention) as the within-subject factor. For variables that did not satisfy the normality assumption, aligned rank transform (ART) ANOVA was performed using the ARTool package in R. ART ANOVA was applied to the non-paretic swing phase and non-paretic stance phase variables. The analyses evaluated the main effects of group and time and the group × time interaction. For the repeated-measures ANOVA, between-group differences in pre–post change and their corresponding 95% confidence intervals were calculated in R based on the ANOVA results. Because the time factor consisted of only two levels (pre- and post-intervention), the assumption of sphericity was not applicable.

When significant interactions were observed, post hoc analyses with Bonferroni correction were performed. Post hoc analyses included within-group pre–post comparisons and between-group comparisons at pre- and post-intervention. Detailed results of the post hoc analyses are provided in Supplementary Tables S1–S5. Effect sizes were calculated using partial eta squared (ηp^2^).

The primary outcomes were prespecified as five outcome constructs: EEG spectral activity (*Theta, SMR,* and *Mid-Beta*), Concentration Index (CI), and Spectral Edge Frequency 50% (SEF-50). The resting, single-task, and dual-task conditions were specified as assessment conditions for these outcomes. Accordingly, multiplicity adjustment was applied at the level of the prespecified primary outcome constructs, rather than treating each condition-specific result as a separate primary outcome. Bonferroni correction was therefore applied across the five prespecified primary outcomes, resulting in an adjusted significance level of α = 0.01 (0.05/5).

A sensitivity analysis using a linear mixed-effects model (LMM) was conducted to assess the robustness of the repeated-measures ANOVA and ART ANOVA findings. In the LMM, Group, Time, and the Group × Time interaction were included as fixed effects, and participant ID was included as a random intercept. A random intercept for participant was included to account for within-participant correlation across time. Models were estimated using restricted maximum likelihood (REML) in IBM SPSS Statistics version 23.0. Estimates, 95% confidence intervals, and *p*-values for the Group × Time interaction were calculated, and the results of the sensitivity analysis are presented in Supplementary Tables S11–S13.

All randomized participants completed the intervention and post-intervention assessments. Therefore, no outcome data were missing and no imputation or other missing-data procedures were required. For analyses other than the primary outcomes, statistical significance was set at *p* < 0.05.

## 3. Results

### 3.1. General Characteristics of the Participants

A total of 61 individuals underwent eligibility screening, of whom 17 were excluded according to the inclusion and exclusion criteria, and the remaining 44 participants were randomly assigned. No participants were lost to follow-up or excluded after randomization, and all 44 participants were randomly assigned in a 1:1 ratio, with 22 participants allocated to each group. Baseline characteristics are presented descriptively in Table 1. No participants were lost to follow-up or excluded after randomization.

No falls, injuries, dizziness, pain, or other adverse events related to the intervention were observed or reported in either group during the 4-week intervention period. Participant-level completion was 100%, as verified using the tablet application connected to the smart walker.

All participants completed all planned intervention sessions and 18 sets per session. Intervention completion was 100%. Performance on the verbal-fluency task was monitored by the therapist during PTCGT sessions but was not quantitatively recorded. The performance of the fruit-naming dual task was monitored in real time by the therapist but was not separately recorded.

### 3.2. Changes in EEG Activity

Significant time × group interaction effects were observed across all prespecified EEG frequency bands (*Theta*, *SMR*, and *Mid-Beta*) (*p* < 0.001), with large interaction estimates (ηp^2^ = 0.775–0.895).

*Theta* power showed large interaction effects under all three conditions—resting, single-task, and dual-task (ηp^2^ = 0.816–0.836)—and tended to be slightly higher during the task conditions than during the resting condition.

For the *SMR* and *Mid-Beta* bands, larger interaction effects were observed under the task-performing conditions, with the largest effects occurring during dual-task walking. In particular, the largest effects were observed under the dual-task condition for SMR (ηp^2^ = 0.878) and *Mid-Beta* (ηp^2^ = 0.895). Because task content and the cumulative addition of task components differed between the experimental protocols, these findings are most appropriately interpreted as protocol-related differences in EEG spectral changes under the tested conditions.

Detailed statistical results are presented in Table 2 and Table S6. The results of the sensitivity analysis using linear mixed-effects models for the EEG-related outcome variables, including *Theta*, *SMR*, *Mid-Beta*, SEF-50, and CI, are presented in Supplementary Table S11.

### 3.3. Changes in EEG Spectral Edge Frequency

Significant time × group interaction effects were also observed for EEG Spectral Edge Frequency 50% (SEF-50) under all conditions (*p* < 0.001).

The effect size was ηp^2^ = 0.677 under the resting condition and increased under the single-task (ηp^2^ = 0.891) and dual-task (ηp^2^ = 0.856) conditions. The largest effect was observed under the single-task condition.

These findings suggest that the two protocols produced different pre- to post-intervention changes in the distribution of scalp-recorded spectral power, particularly during task performance. Detailed statistical results are presented in Table 3 and Table S7.

### 3.4. Changes in Concentration Index

Significant time × group interaction effects were observed for the Concentration Index (CI) under all conditions (*p* < 0.001). The effect sizes ranged from ηp^2^ = 0.460 to 0.804, representing the largest magnitude among the variables examined in this study.

Because CI is a composite spectral ratio rather than a validated direct measure of concentration, these findings are interpreted as protocol-related changes in an EEG spectral index.

The largest interaction effect was observed under the dual-task condition (ηp^2^ = 0.804). This indicates that the magnitude of the pre–post difference between the protocols was greatest during dual-task walking; however, this finding alone does not demonstrate improved cognitive efficiency or attentional resource allocation.

Detailed results are presented in Table 4 and Table S8.

### 3.5. Changes in Dual-Task Cost

A significant time × group interaction effect was also observed for Dual-Task Cost (DTC) (F = 4.433, *p* = 0.041), with an effect size of ηp^2^ = 0.095.

The group × time interaction was statistically significant (F = 4.433, *p* = 0.041, ηp^2^ = 0.095).

However, in the sensitivity analysis using a linear mixed-effects model, the same interaction was not statistically significant (*p* = 0.088).

Detailed results are presented in Table 5 and Table S9. The results of the sensitivity analysis using a linear mixed-effects model are presented in Supplementary Table S12.

### 3.6. Changes in Gait Performance

Significant time × group interaction effects were observed across all gait-related variables (*p* < 0.001), with large to very large interaction effects (ηp^2^ = 0.784–0.987). The largest interaction effects were observed for paretic lower-limb variables, including the single-support cycle (ηp^2^ = 0.987), stance phase (ηp^2^ = 0.978), and swing phase (ηp^2^ = 0.976). Very large interaction effects were also observed for stride length (ηp^2^ = 0.973) and gait velocity (ηp^2^ = 0.958). Given the large magnitude of the interaction effects, these findings should be interpreted cautiously because the gait variables are closely interrelated.

Detailed statistical results are presented in Table 6 and Table S10. The results of the sensitivity analysis using a linear mixed-effects model are presented in Supplementary Table S13.

## 4. Discussion

This study compared the effects of two multi-component cognitive–motor gait training protocols using the same sensor-based auditory feedback walker and obstacle-based walking environment. The Progressive Task-Complexity Gait Training group (PTCGT) showed greater pre- to post-intervention changes than the Constant Task-Complexity Gait Training group (CTCGT) in several EEG spectral characteristics and spatiotemporal gait variables. These differences should be understood in the context of the intervention characteristics, as PTCGT was structured to progressively vary task complexity, whereas CTCGT repeatedly performed the same task configuration consisting of metronome cueing and obstacle avoidance. In particular, because PTCGT included an additional verbal fluency dual-task that was not included in CTCGT, the findings are more appropriately interpreted as differences between the two multi-component cognitive–motor gait training protocols tested rather than as evidence of an independent effect of a specific sequential progression strategy.

### 4.1. Changes in EEG Spectral Characteristics and EEG-Derived Measures

The present study observed a reduction in *Theta* power and increases in *SMR* and *Mid-Beta* power after training, with these changes being particularly evident under the dual-task condition. Changes were also observed in CI and SEF-50. These concurrent changes across multiple EEG measures may be understood as changes in overall EEG spectral characteristics during gait performance after training rather than as independent changes in individual frequency bands [25].

Although *Theta*, *SMR*, and *Beta* band activity have been associated with cognitive processing, attentional control, and sensorimotor-related processes, the functional significance of each frequency band may vary according to task characteristics and analytical methods [26,27]. In addition, dual-task walking in individuals with stroke has been reported to involve multiple regions, including the prefrontal, premotor, sensorimotor, and parietal areas [28]. Therefore, EEG changes observed during walking are difficult to explain on the basis of a single frequency band or a specific brain region alone.

The concurrent reduction in *Theta* and increases in *SMR* and *Mid-Beta* observed in the present study indicate the possibility of a change in the EEG spectral pattern associated with cognitive–motor gait performance after training. The fact that these changes were more pronounced under the dual-task condition suggests that differences in EEG changes between the two protocols were more evident under conditions involving multiple concurrent task components. However, because the present study used whole-scalp average spectral power, these changes cannot be attributed to a specific cortical region or neural network.

Differences between the protocols were also observed in CI and SEF-50. CI reflects the relative power relationship between *SMR* and *Mid-Beta* and *Theta* [22], whereas SEF-50 summarizes the distribution of spectral power across the overall frequency range [29]. Therefore, changes in these indices may be understood as findings indicating changes in EEG spectral distribution after training together with the changes observed in individual frequency bands. However, these changes alone cannot directly demonstrate improvements in cognitive efficiency, attentional resource allocation, or gait automaticity. In particular, because the accuracy, number of responses, and errors in the cognitive task performed during dual-task walking were not measured in the present study, it cannot be determined whether the EEG changes were associated with actual improvement in cognitive performance.

In addition, the use of mobile EEG during walking requires consideration of the potential influence of movement-related artifacts. In the present study, filtering, ICA-based artifact correction, and epoch rejection based on amplitude criteria were applied, and an average of 29% of the collected epochs were excluded during preprocessing. However, because the influence of changes in walking patterns before and after the intervention on EEG signals cannot be completely excluded, the EEG findings of the present study are more appropriately interpreted as changes in EEG spectral characteristics after training rather than direct evidence of a specific neural mechanism.

### 4.2. Changes in Cognitive–Motor Interference and Gait Performance

The PTCGT group showed greater pre- to post-intervention changes than the CTCGT group across several spatiotemporal gait variables. Gait velocity and cadence increased, as did step length and stride length, and changes were also observed in single-support and stance-related variables of the paretic lower limb. These findings may be understood as a pattern of concurrent changes in multiple spatiotemporal components related to forward progression, step length, and lower-limb support rather than as a change limited to a single gait measure.

Gait velocity increased by approximately 0.161 m/s in the PTCGT group and 0.081 m/s in the CTCGT group. Although a clinically meaningful change of approximately 0.16 m/s has been reported for gait velocity in individuals with stroke [30], such thresholds may vary according to the stage of recovery and functional level of the participants. In addition, the characteristics of participants in previous studies were not completely identical to those of the chronic stroke sample in the present study. Therefore, the within-group change of 0.161 m/s observed in PTCGT may be considered comparable to a previously reported threshold for clinically meaningful change; however, the between-group difference in change was approximately 0.080 m/s, and this difference should be interpreted cautiously rather than as definitive clinical superiority.

In addition to gait velocity, the PTCGT group showed a 10.36-cm increase in paretic step length, compared with a 3.91-cm increase in the CTCGT group. A previous study reported a minimal detectable change (MDC) of approximately 6.75 cm for paretic step length in individuals with chronic stroke [31]. Thus, the change observed in PTCGT exceeded this previously reported measurement-error threshold, whereas the change in CTCGT did not. However, because the previous estimate was obtained under different gait assessment conditions, this comparison should be regarded as a reference for interpreting the magnitude of change rather than as direct evidence of clinical meaningfulness.

The concurrent changes observed across several gait variables indicate a broader change in spatiotemporal gait patterns that cannot be explained solely by an increase in gait velocity. In particular, the changes in paretic-limb support-related variables occurring together with changes in gait velocity and step length suggest that temporal characteristics of weight support and lower-limb gait may have changed after training. However, because these gait variables are closely interrelated, the significant findings for individual variables should not be interpreted as independent treatment effects but rather as a pattern of concurrent changes across related gait components.

The very large partial ηp^2^ values observed for several of these outcomes should also be interpreted cautiously. These estimates may partly reflect the repeated-measures design, which reduces between-participant variability by accounting for within-participant changes, as well as the relatively low within-group variability observed in several outcomes. In addition, the strong physiological and mathematical interdependence among spatiotemporal gait variables may have contributed to the consistently large interaction estimates across related gait measures. Therefore, the magnitude of ηp^2^ should not be interpreted as evidence of equivalently large independent treatment effects across each gait variable. Replication in independent samples will be important to determine the stability of these estimates.

Although a greater reduction in DTC was observed in PTCGT, the group × time interaction was statistically significant in the primary analysis but did not remain statistically significant in the linear mixed-effects model sensitivity analysis. Therefore, the between-group difference in DTC should be interpreted as a less statistically robust finding than the other gait outcomes. In addition, because DTC in the present study was calculated using walking time alone, the accuracy or response rate of cognitive-task performance could not be considered. Thus, the DTC findings should be interpreted as an auxiliary outcome reflecting changes in gait-related cost under dual-task conditions rather than as evidence of an overall improvement in dual-task ability [32].

### 4.3. Clinical Implications

The changes observed in the present study suggest that, even when the same walker and walking environment are used, the way in which cognitive–motor tasks are configured and their complexity is organized may be associated with different patterns of changes in gait performance and EEG spectral characteristics after training. In particular, the PTCGT group showed concurrent changes across several spatiotemporal gait variables, including gait velocity, step length, and lower-limb support-related characteristics. These findings suggest that changes in gait performance after training may have involved multiple gait components rather than simply an increase in walking speed.

The clinical relevance of these changes lies in the fact that gait performance is not determined by a single measure. Concurrent changes in gait velocity, step length, and lower-limb support time may be related to changes in how individuals move the body forward and support body weight on the paretic lower limb during walking. However, because the present study did not directly assess mobility in daily life, falls, activity, or participation, it cannot be determined whether these gait changes resulted in improved functional independence in real-world situations.

Importantly, the clinical interpretation of these findings should also take into account that the magnitude of change differed across gait variables and that not all statistically significant changes necessarily represent clinically meaningful improvement. The gait velocity change in PTCGT was comparable to a previously reported MCID reference, whereas the between-group difference in change was smaller than this threshold. Similarly, the increase in paretic step length in PTCGT exceeded a previously reported MDC, but this does not by itself establish a patient-perceived functional benefit.

Therefore, the findings of the present study should be interpreted not as evidence that a specific stepwise training strategy is clinically superior, but as preliminary evidence that the multi-component cognitive–motor gait training protocols tested in this study may induce changes in gait performance and EEG spectral characteristics in individuals with chronic stroke. Future studies should evaluate patient-centered outcomes, including mobility in daily life, falls, activity, and participation, in addition to gait velocity and spatiotemporal gait variables.

### 4.4. Limitations

This study has several limitations.

First, the intervention components of the two training protocols were not completely identical. PTCGT introduced an additional verbal fluency dual-task at the final stage while progressively increasing task complexity, whereas CTCGT did not include this task. Therefore, between-group differences cannot be attributed solely to the sequential progression of task demands, and the effects of additional cognitive-task exposure, task complexity, and task specificity may have also contributed.

Second, the participants were relatively high-functioning individuals with chronic stroke who had MMSE-K scores ≥ 24, FAC scores ≥ 3, sufficient balance to stand without support, and adequate upper-extremity function for walker use. Therefore, the findings may have limited generalizability to individuals with cognitive impairment, more severe gait dysfunction, aphasia, or acute or subacute stroke.

Third, cognitive-task performance during dual-task walking was not directly measured. Therefore, improvements in cognitive–motor integration or attentional resource allocation cannot be demonstrated solely from changes in DTC or EEG measures, and the possibility that participants changed their prioritization between walking and the cognitive task cannot be excluded.

Fourth, EEG analysis was based on whole-scalp average spectral measures and therefore did not directly assess activity in specific cortical regions, functional connectivity, or network-level reorganization. In addition, mobile EEG during walking may be affected by movement and muscle artifacts, and the influence of residual artifacts cannot be completely excluded despite the application of artifact-correction procedures.

Fifth, although both groups continued their usual inpatient rehabilitation program during the study period, the detailed content and treatment dose of these interventions were not fully standardized or controlled as part of the experimental protocol. Therefore, differences in concurrent rehabilitation may have partially influenced the outcomes.

Finally, large effect sizes were observed for several outcomes, and the spatiotemporal gait variables are highly interdependent. Although mean changes and confidence intervals were additionally presented and a sensitivity analysis using a linear mixed-effects model was conducted, the robustness of these effect estimates requires further confirmation in larger and independent studies.

## 5. Conclusions

The PTCGT group showed greater pre- to post-intervention changes than the CTCGT group in several EEG spectral characteristics, including decreased *Theta* and increased *SMR* and *Mid-Beta* activity, as well as in CI and SEF-50. Greater changes were also observed across several spatiotemporal gait variables in PTCGT than in CTCGT. These differences were particularly evident under dual-task conditions and suggest that the specific multi-component cognitive–motor gait training protocols tested in this study may be associated with changes in EEG spectral characteristics and gait performance.

However, because the two training protocols differed not only in task progression but also in task configuration and cumulative exposure to cognitive tasks, the observed between-group differences cannot be attributed to an independent effect of progressive task sequencing. In addition, the between-group difference in DTC did not retain the same statistical significance in the sensitivity analysis; therefore, the DTC findings should be interpreted cautiously. Future studies should more rigorously control the intervention components and cumulative cognitive-task exposure, quantify cognitive-task performance during dual-task walking, and use long-term follow-up and network-level neurophysiological analyses to further determine the clinical significance and underlying mechanisms of these findings.

## Figures and Tables

**Table 1:** General characteristics of the subjects (*N* = 44).

Variable	PTCGT ^a^ (*n* = 22)	CTCGT ^b^ (*n* = 22)
Age (year)	63.45 ± 7.39	60.63 ± 7.57
Gender (Male/Female)	12/10	10/12
Onset (months)	16.54 ± 4.51	15.72 ± 5.40
Diagnosis (Infarction/Hemorrhage)	16/6	16/6
Affected side (Left/Right)	13/9	10/12
Height (cm)	167.45 ± 8.17	166.22 ± 9.28
Weight (kg)	66.77 ± 9.59	63.40 ± 9.05
MMSE-K ^c^ (score)	26.09 ± 1.26	26.27 ± 1.24

^a^. PTCGT: Progressive Task-Complexity Gait Training group; ^b^. CTCGT: Constant Task-Complexity Gait Training group; ^c^. MMSE-K: Korean Mini-Mental State Examination.

**Table 2:** Changes in EEG spectral measures before and after intervention (*N* = 44).

Variable		Group	Pre (Mean ± SD ^c^)	Post (Mean ± SD ^c^)	Change (Mean ± SD ^c^)	Between-Group Difference in Change (95% CI ^d^)	Time × Group (F, *p*)	ηp^2 e^
*Theta*	Rest	PTCGT ^a^	19.22 ± 1.37	18.49 ± 1.18	−0.73 ± 0.20	−0.73 (−0.82 to −0.63)	F = 186.151, *p* < 0.001 ^†^	0.816
		CTCGT ^b^	19.28 ± 1.43	19.28 ± 1.37	0.00 ± 0.09			
	Single	PTCGT ^a^	17.65 ± 1.24	16.42 ± 0.84	−1.23 ± 0.41	−1.07 (−1.25 to −0.89)	F = 213.951, *p* < 0.001 ^†^	0.836
		CTCGT ^b^	17.69 ± 1.11	17.53 ± 1.06	−0.15 ± 0.07			
	Dual	PTCGT ^a^	20.48 ± 1.82	18.10 ± 1.13	−2.37 ± 0.70	−2.08 (−2.39 to −1.77)	F = 208.542, *p* < 0.001 ^†^	0.832
		CTCGT ^b^	20.55 ± 1.74	20.25 ± 1.61	0.29 ± 0.16			
*SMR*	Rest	PTCGT ^a^	8.30 ± 0.67	8.79 ± 0.80	0.49 ± 0.13	0.41 (0.35 to 0.47)	F = 182.989, *p* < 0.001 ^†^	0.813
		CTCGT ^b^	8.31 ± 0.68	8.39 ± 0.69	0.08 ± 0.03			
	Single	PTCGT ^a^	9.50 ± 0.79	10.75 ± 1.06	1.24 ± 0.27	1.12 (1.04 to 1.25)	F = 252.148, *p* < 0.001 ^†^	0.857
		CTCGT ^b^	9.50 ± 0.78	9.61 ± 0.80	0.11 ± 0.07			
	Dual	PTCGT ^a^	10.30 ± 0.96	12.08 ± 1.28	1.77 ± 0.03	1.67 (1.52 to 1.81)	F = 302.550, *p* < 0.001 ^†^	0.878
		CTCGT ^b^	10.32 ± 0.97	10.43 ± 1.00	0.10 ± 0.06			
*Mid* *-* *Beta*	Rest	PTCGT ^a^	7.47 ± 0.66	8.00 ± 0.81	0.53 ± 0.16	0.45 (0.38 to 0.53)	F = 144.895, *p* < 0.001 ^†^	0.775
		CTCGT ^b^	7.46 ± 0.67	7.54 ± 0.69	0.07 ± 0.06			
	Single	PTCGT ^a^	8.42 ± 0.86	9.85 ± 1.18	1.43 ± 0.34	1.33 (1.18 to 1.48)	F = 250.389, *p* < 0.001 ^†^	0.856
		CTCGT ^b^	8.37 ± 0.82	8.47 ± 0.85	0.09 ± 0.05			
	Dual	PTCGT ^a^	9.46 ± 1.06	11.50 ± 1.39	2.04 ± 0.35	1.96 (1.80 to 2.11)	F = 359.743, *p* < 0.001 ^†^	0.895
		CTCGT ^b^	9.44 ± 1.06	9.52 ± 1.07	0.08 ± 0.03			

^a^. PTCGT: Progressive Task-Complexity Gait Training group; ^b^. CTCGT: Constant Task-Complexity Gait Training group; ^c^. SD: Standard Deviation; ^d^. CI: Confidence Interval; ^e^. ηp^2^: partial eta squared. ^†^ *p* < 0.01, Bonferroni-adjusted significance threshold for the five primary outcomes. The 95% CIs were computed for the between-group difference in individual change scores (post–pre) using R.

**Table 3:** Changes in Spectral Edge Frequency 50% (SEF-50) before and after intervention (*N* = 44).

Variable	Group	Pre (Mean ± SD ^c^)	Post (Mean ± SD ^c^)	Change (Mean ± SD ^c^)	Between-Group Difference in Change (95% CI ^d^)	Time × Group (F, *p*)	ηp^2 e^
Rest	PTCGT ^a^	9.57 ± 0.35	9.86 ± 0.42	0.29 ± 0.75	0.20 (0.15 to 0.25)	F = 87.931, *p* < 0.001 ^†^	0.677
	CTCGT ^b^	9.57 ± 0.35	9.66 ± 0.39	0.08 ± 0.08			
Single	PTCGT ^a^	10.15 ± 0.41	10.89 ± 0.51	0.73 ± 0.12	0.65 (0.60 to 0.71)	F = 342.080, *p* < 0.001 ^†^	0.891
	CTCGT ^b^	10.17 ± 0.39	10.25 ± 0.41	0.07 ± 0.04			
Dual	PTCGT ^a^	10.84 ± 0.47	11.60 ± 0.54	0.76 ± 0.89	0.69 (0.64 to 0.73)	F = 250.613, *p* < 0.001 ^†^	0.856
	CTCGT ^b^	10.85 ± 0.49	10.93 ± 0.50	0.07 ± 0.04			

^a^. PTCGT: Progressive Task-Complexity Gait Training group; ^b^. CTCGT: Constant Task-Complexity Gait Training group; ^c^. SD: Standard Deviation; ^d^. CI: Confidence Interval; ^e^. ηp^2^: partial eta squared. ^†^ *p* < 0.01, Bonferroni-adjusted significance threshold for the five primary outcomes. The 95% CIs were computed for the between-group difference in individual change scores (post–pre) using R.

**Table 4:** Changes in Concentration Index (CI) before and after intervention (*N* = 44).

Variable	Group	Pre (Mean ± SD ^c^)	Post (Mean ± SD ^c^)	Change (Mean ± SD ^c^)	Between-Group Difference in Change (95% CI ^d^)	Time × Group (F, *p*)	ηp^2 e^
Rest	PTCGT ^a^	0.81 ± 0.03	0.94 ± 0.08	0.13 ± 0.04	0.08 (0.05 to 0.11)	F = 35.818, *p* < 0.001 ^†^	0.460
	CTCGT ^b^	0.83 ± 0.04	0.88 ± 0.22	0.05 ± 0.06			
Single	PTCGT ^a^	1.04 ± 0.08	1.25 ± 0.07	0.21 ± 0.07	0.20 (0.16 to 0.24)	F = 94.920, *p* < 0.001 ^†^	0.693
	CTCGT ^b^	1.03 ± 0.13	1.04 ± 0.08	0.01 ± 0.05			
Dual	PTCGT ^a^	0.97 ± 0.08	1.30 ± 0.08	0.33 ± 0.09	0.28 (0.24 to 0.32)	F = 172.629, *p* < 0.001 ^†^	0.804
	CTCGT ^b^	0.94 ± 0.09	0.99 ± 0.07	0.05 ± 0.07			

^a^. PTCGT: Progressive Task-Complexity Gait Training group; ^b^. CTCGT: Constant Task-Complexity Gait Training group; ^c^. SD: Standard Deviation; ^d^. CI: Confidence Interval; ^e^. ηp^2^: partial eta squared. ^†^ *p* < 0.01, Bonferroni-adjusted significance threshold for the five primary outcomes. The 95% CIs were computed for the between-group difference in individual change scores (post–pre) using R.

**Table 5:** Changes in Dual-Task Cost (DTC) before and after intervention (*N* = 44).

Variable	Group	Pre (Mean ± SD ^c^)	Post (Mean ± SD ^c^)	Change (Mean ± SD ^c^)	Between-Group Difference in Change (95% CI ^d^)	Time × Group (F, *p*)	ηp^2 e^
DTC ^f^ (%)	PTCGT ^a^	14.17 ± 5.21	8.75 ± 3.46	−5.41 ± 6.65	−3.21 (−7.08 to 0.55)	F = 4.433, *p* = 0.041	0.095
	CTCGT ^b^	15.41 ± 4.12	13.26 ± 3.41	−2.21 ± 5.87			

^a^. PTCGT: Progressive Task-Complexity Gait Training group; ^b^. CTCGT: Constant Task-Complexity Gait Training group; ^c^. SD: Standard Deviation; ^d^. CI: Confidence Interval; ^e^. ηp^2^: partial eta squared; ^f^. DTC: Dual-Task Cost. The 95% CIs were computed for the between-group difference in individual change scores (post–pre) using R.

**Table 6:** Changes in spatiotemporal gait variables assessed using GAITRite before and after intervention (*N* = 44).

Variable	Group	Pre (Mean ± SD ^c^)	Post (Mean ± SD ^c^)	Change (Mean ± SD ^c^)	Between-Group Difference in Change (95% CI ^d^)	Time × Group (F, *p*)	ηp^2 e^
Velocity (cm/s)	PTCGT ^a^	56.24 ± 2.55	72.30 ± 2.70	16.06 ± 0.95	8.01 (7.58 to 8.43)	F = 952.550, *p* < 0.001	0.958
	CTCGT ^b^	55.41 ± 1.44	63.47 ± 1.39	8.05 ± 0.21			
Cadence (steps/min)	PTCGT ^a^	85.45 ± 3.01	97.20 ± 2.80	11.75 ± 1.06	6.84 (6.37 to 7.31)	F = 556.208, *p* < 0.001	0.930
	CTCGT ^b^	84.47 ± 1.74	89.38 ± 1.52	4.91 ± 0.26			
Stride length (cm)	PTCGT ^a^	74.15 ± 3.45	94.31 ± 3.48	20.15 ± 1.07	12.00 (11.53 to 12.46)	F = 1540.831, *p* < 0.001	0.973
	CTCGT ^b^	72.99 ± 1.99	81.14 ± 2.07	8.15 ± 0.10			
Paretic Step Length (cm)	PTCGT ^a^	37.55 ± 2.36	47.91 ± 2.04	10.36 ± 0.79	6.44 (6.10 to 6.79)	F = 888.580, *p* < 0.001	0.955
	CTCGT ^b^	36.76 ± 1.23	40.68 ± 1.10	3.91 ± 0.16			
Non-paretic Step Length (cm)	PTCGT ^a^	45.62 ± 2.10	49.80 ± 1.86	4.18 ± 0.57	2.55 (2.30 to 2.81)	F = 406.543, *p* < 0.00	0.906
	CTCGT ^b^	44.93 ± 1.28	46.56 ± 1.16	0.62 ± 0.15			
Paretic Swing (%)	PTCGT ^a^	32.31 ± 1.31	38.04 ± 1.18	5.73 ± 0.30	4.36 (4.22 to 4.50)	F = 1692.516, *p* < 0.001	0.976
	CTCGT ^b^	31.77 ± 0.71	33.14 ± 0.66	1.37 ± 0.11			
Non-paretic Swing (%)	PTCGT ^a^	38.67 ± 1.12	40.37 ± 0.94	1.69 ± 0.28	0.92 (0.80 to 1.04)	F = 154.814, *p* < 0.001	0.787
	CTCGT ^b^	38.12 ± 0.55	38.90 ± 0.54	0.77 ± 0.03			
Paretic Stance (%)	PTCGT ^a^	67.76 ± 1.28	61.98 ± 1.17	5.78 ± 0.28	4.41 (−4.54 to −4.28)	F = 1857.606, *p* < 0.001	0.978
	CTCGT ^b^	68.23 ± 0.71	66.86 ± 0.66	−1.37 ± 0.11			
Non-paretic Stance (%)	PTCGT ^a^	61.33 ± 1.12	59.63 ± 0.94	−1.69 ± 0.28	−0.92 (−1.04 to −0.79)	F = 152.578, *p* < 0.001	0.784
	CTCGT ^b^	61.90 ± 0.54	61.12 ± 0.54	−0.77 ± 0.36			
Paretic Single Support cycle (%)	PTCGT ^a^	26.18 ± 1.31	32.51 ± 1.46	6.32 ± 0.40	4.98 (4.81 to 5.16)	F = 3176.245, *p* < 0.001	0.987
	CTCGT ^b^	25.73 ± 0.78	27.06 ± 0.73	1.33 ± 0.07			
Non-paretic Single Support cycle (%)	PTCGT ^a^	31.87 ± 1.31	35.76 ± 1.36	3.89 ± 0.30	2.63 (2.50 to 2.77)	F = 525.229, *p* < 0.001	0.926
	CTCGT ^b^	31.36 ± 0.70	32.61 ± 0.72	1.25 ± 0.04			
Double Support cycle (%)	PTCGT ^a^	29.85 ± 1.56	20.63 ± 1.33	−9.21 ± 0.52	−5.60 (−5.87 to −5.33)	F = 1725.857, *p* < 0.001	0.976
	CTCGT ^b^	30.42 ± 0.94	26.81 ± 0.61	−3.61 ± 0.35			

^a^. PTCGT: Progressive Task-Complexity Gait Training group; ^b^. CTCGT: Constant Task-Complexity Gait Training group; ^c^. SD: Standard Deviation; ^d^. CI: Confidence Interval; ^e^. ηp^2^: partial eta squared. The 95% CIs were computed for the between-group difference in individual change scores (post–pre) using R.

## Data Availability

The data presented in this study are available from the corresponding author upon reasonable request.

## Supplementary Materials

The following supporting information can be downloaded at: https://www.mdpi.com/article/10.3390/medicina62091738/s1, Table S1: Bonferroni-adjusted Post Hoc Pairwise Comparisons for EEG Spectral Measures; Table S2: Bonferroni-adjusted Post Hoc Pairwise Comparisons for Spectral Edge Frequency 50% (SEF-50); Table S3: Bonferroni-adjusted Post Hoc Pairwise Comparisons for Concentration Index (CI); Table S4: Bonferroni-adjusted Post Hoc Pairwise Comparisons for Dual-Task Cost (DTC); Table S5: Bonferroni-adjusted Post Hoc Pairwise Comparisons for Spatiotemporal Gait Variables Assessed Using GAITRite; Table S6: Changes in electroencephalography (EEG) power spectrum variables before and after intervention (*N* = 44); Table S7: Changes in Spectral Edge Frequency 50% (SEF-50) before and after intervention (*N* = 44); Table S8: Changes in Concentration Index (CI) before and after intervention (*N* = 44); Table S9: Changes in Dual-Task Cost (DTC) before and after intervention (*N* = 44); Table S10: Changes in Spatiotemporal Gait Variables Assessed Using GAITRite before and after intervention (*N* = 44); Table S11: Sensitivity Analysis Using Linear Mixed-Effects Models for Group × Time Interactions in EEG-Derived Outcomes; Table S12: Sensitivity Analysis Using a Linear Mixed-Effects Model for the Group × Time Interaction in Dual-Task Cost (DTC); Table S13: Sensitivity Analysis Using Linear Mixed-Effects Models for Group × Time Interactions in Spatiotemporal Gait Variables.
