# Supplementary material for: Effects of Two Different Multicomponent Cognitive–Motor Training Protocols on EEG Spectral Activity, Cognitive–Motor Interference, and Gait Performance in Individuals with Chronic Stroke: A Randomized Controlled Trial
Source: Medicina (Kaunas). 2026 Sep 9;62(9):1738. doi: 10.3390/medicina62091738 (PMC13609261; doi:10.3390/medicina62091738)
Supplement: Supplementary file 1 [file medicina-62-01738-s001.zip › medicina-4547507-supplementary.pdf]

Table S1. Bonferroni-adjusted Post Hoc Pairwise Comparisons for EEG Spectral Measures

Variable		Comparison	Mean Difference	95% CI [c]	p-value
Theta	Rest	PTCGT [a] Pre vs Post	0.73	0.64 to 0.88	< 0.001
		CTCGT [b] Pre vs Post	0.00	-0.04 to 0.04	1.000
		PTCGT [a] Pre vs CTCGT [b] Pre	-0.55	-0.90 to 0.80	0.898
		PTCGT [a] Post vs CTCGT [b] Post	-0.78	-1.54 to -0.06	0.048
	Single	PTCGT [a] Pre vs Post	1.23	1.04 to 1.47	< 0.001
		CTCGT [b] Pre vs Post	0.159	0.12 to 0.19	0.067
		PTCGT [a] Pre vs CTCGT [b] Pre	-0.32	-0.75 to 0.68	1.000
		PTCGT [a] Post vs CTCGT [b] Post	-1.10	-1.68 to -0.52	0.007
	Dual	PTCGT [a] Pre vs Post	2.37	2.06 to 2.69	< 0.001
		CTCGT [b] Pre vs Post	0.29	0.22 to 0.36	0.118
		PTCGT [a] Pre vs CTCGT [b] Pre	-0.06	-1.15 to -1.05	1.000
		PTCGT [a] Post vs CTCGT [b] Post	-2.15	-2.99 to -1.30	< 0.001
SMR	Rest	PTCGT [a] Pre vs Post	0.49	0.43 to 0.55	< 0.001
		CTCGT [b] Pre vs Post	0.08	0.06 to 0.09	< 0.001
		PTCGT [a] Pre vs CTCGT [b] Pre	-0.01	-0.42 to 0.39	0.947
		PTCGT [a] Post vs CTCGT [b] Post	0.40	-0.05 to 0.85	0.084
	Single	PTCGT [a] Pre vs Post	1.24	1.11 to 1.36	< 0.001
		CTCGT [b] Pre vs Post	0.11	0.08 to 0.14	< 0.001
		PTCGT [a] Pre vs CTCGT [b] Pre	0.00	-0.46 to 0.46	0.970
		PTCGT [a] Post vs CTCGT [b] Post	1.36	0.56 to 1.70	< 0.001
	Dual	PTCGT [a] Pre vs Post	1.77	1.62 to 1.92	< 0.001
		CTCGT [b] Pre vs Post	0.10	1.17 to 0.13	< 0.001
		PTCGT [a] Pre vs CTCGT [b] Pre	-0.02	-0.60 to 0.56	0.938
		PTCGT [a] Post vs CTCGT [b] Post	1.65	0.95 to 2.35	< 0.001
Mid-beta	Rest	PTCGT [a] Pre vs Post	0.53	0.46 to 0.61	< 0.001
		CTCGT [b] Pre vs Post	0.07	0.04 to 0.10	< 0.001
		PTCGT [a] Pre vs CTCGT [b] Pre	0.00	-0.39 to 0.40	0.982
		PTCGT [a] Post vs CTCGT [b] Post	0.46	0.06 to 0.92	0.047
	Single	PTCGT [a] Pre vs Post	1.43	1.28 to 1.58	< 0.001
		CTCGT [b] Pre vs Post	0.09	0.00 to 0.12	< 0.001

	PTCGT [a] Pre vs CTCGT [b] Pre	0.04	-0.46 to 0.55	0.860
	PTCGT [a] Post vs CTCGT [b] Post	1.38	0.75 to 2.00	< 0.001
Dual	PTCGT [a] Pre vs Post	2.04	1.88 to 2.20	< 0.001
	CTCGT [b] Pre vs Post	0.08	0.06 to 0.09	< 0.001
	PTCGT [a] Pre vs CTCGT [b] Pre	0.01	-0.62 to 0.66	0.955
	PTCGT [a] Post vs CTCGT [b] Post	1.98	1.22 to 2.73	< 0.001

a. PTCGT: Progressive Task-Complexity Gait Training group; b. CTCGT: Constant Task-Complexity Gait Training group; c. CI: Confidence Interval. The mean differences and their 95% CIs were computed in SPSS for the pairwise comparisons specified in the Comparison column, including within-group pre–post and between-group pre- or post-intervention comparisons.

Table S2. Bonferroni-adjusted Post Hoc Pairwise Comparisons for Spectral Edge Frequency 50% (SEF-50)

Variable	Comparison	Mean Difference	95% CI [c]	p-value
Rest	PTCGT [a] Pre vs Post	0.29	0.25 to 0.32	< 0.001
	CTCGT [b] Pre vs Post	0.08	0.04 to 0.12	< 0.001
	PTCGT [a] Pre vs CTCGT [b] Pre	-0.00	-0.21 to 0.20	0.966
	PTCGT [a] Post vs CTCGT [b] Post	0.20	-0.04 to 0.44	0.109
Single	PTCGT [a] Pre vs Post	0.73	0.67 to 0.78	< 0.001
	CTCGT [b] Pre vs Post	0.07	0.05 to 0.09	< 0.001
	PTCGT [a] Pre vs CTCGT [b] Pre	-0.01	-0.26 to 0.22	0.881
	PTCGT [a] Post vs CTCGT [b] Post	0.64	0.35 to 0.92	< 0.001
Dual	PTCGT [a] Pre vs Post	0.76	0.72 to 0.80	< 0.001
	CTCGT [b] Pre vs Post	0.07	0.05 to 0.09	< 0.001
	PTCGT [a] Pre vs CTCGT [b] Pre	-0.01	-0.31 to 0.27	0.901
	PTCGT [a] Post vs CTCGT [b] Post	0.67	0.35 to 0.98	< 0.001

a. PTCGT: Progressive Task-Complexity Gait Training group; b. CTCGT: Constant Task-Complexity Gait Training group; c. CI: Confidence Interval. The mean differences and their 95% CIs were computed in SPSS for the pairwise comparisons specified in the Comparison column, including within-group pre–post and between-group pre- or post-intervention comparisons.

Table S3. Bonferroni-adjusted Post Hoc Pairwise Comparisons for Concentration Index (CI)

Variable	Comparison	Mean Difference	95% CI [c]	p-value
Rest	PTCGT [a] Pre vs Post	0.13	0.11 to 0.14	< 0.001
	CTCGT [b] Pre vs Post	0.05	0.02 to 0.07	< 0.001
	PTCGT [a] Pre vs CTCGT [b] Pre	-0.02	-0.04 to 0.00	0.068
	PTCGT [a] Post vs CTCGT [b] Post	0.06	-0.04 to 0.16	0.240
Single	PTCGT [a] Pre vs Post	0.21	0.17 to 0.24	< 0.001
	CTCGT [b] Pre vs Post	0.01	-0.01 to 0.03	0.359
	PTCGT [a] Pre vs CTCGT [b] Pre	0.01	-0.05 to 0.07	0.760
	PTCGT [a] Post vs CTCGT [b] Post	0.21	0.16 to 0.25	< 0.001
Dual	PTCGT [a] Pre vs Post	0.33	0.29 to 0.37	< 0.001
	CTCGT [b] Pre vs Post	0.05	0.01 to 0.08	0.003
	PTCGT [a] Pre vs CTCGT [b] Pre	0.03	0.02 to 0.08	0.249
	PTCGT [a] Post vs CTCGT [b] Post	0.31	0.26 to 0.35	< 0.001

a. PTCGT: Progressive Task-Complexity Gait Training group; b. CTCGT: Constant Task-Complexity Gait Training group; c. CI: Confidence Interval. The mean differences and their 95% CIs were computed in SPSS for the pairwise comparisons specified in the Comparison column, including within-group pre–post and between-group pre- or post-intervention comparisons.

Table S4. Bonferroni-adjusted Post Hoc Pairwise Comparisons for Dual-Task Cost (DTC)

Variable	Comparison	Mean Difference	95% CI [c]	p-value
DTC [d]	PTCGT [a] Pre vs Post	0.34	0.29 to 0.38	< 0.001
	CTCGT [b] Pre vs Post	0.03	-0.04 to 0.04	0.898
	PTCGT [a] Pre vs CTCGT [b] Pre	-0.01	-0.06 to 0.03	0.498
	PTCGT [a] Post vs CTCGT [b] Post	0.31	0.27 to 0.36	< 0.001

a. PTCGT: Progressive Task-Complexity Gait Training group; b. CTCGT: Constant Task-Complexity Gait Training group; c. CI: Confidence Interval; d. DTC: Dual-Task Cost. The mean differences and their 95% CIs were computed in SPSS for the pairwise comparisons specified in the Comparison column, including within-group pre–post and between-group pre- or post-intervention comparisons.

Table S5. Bonferroni-adjusted Post Hoc Pairwise Comparisons for Spatiotemporal Gait Variables Assessed Using GAITRite

Variable	Comparison	Mean Difference	95% CI [c]	p-value
Velocity (cm/s)	PTCGT [a] Pre vs Post	16.06	15.642 to 16.493	< 0.001
	CTCGT [b] Pre vs Post	8.05	7.960 to 8.151	< 0.001
	PTCGT [a] Pre vs CTCGT [b] Pre	0.82	−0.448 to 2.098	0.202
	PTCGT [a] Post vs CTCGT [b] Post	8.83	7.515 to 10.159	< 0.001
Cadence (steps/min)	PTCGT [a] Pre vs Post	11.75	11.289 to 12.230	< 0.001
	CTCGT [b] Pre vs Post	4.91	4.797 to 5.028	< 0.001
	PTCGT [a] Pre vs CTCGT [b] Pre	0.97	−0.534 to 2.491	0.201
	PTCGT [a] Post vs CTCGT [b] Post	7.82	6.441 to 9.210	< 0.001
Stride Length (cm)	PTCGT [a] Pre vs Post	20.16	19.684 to 20.635	< 0.001
	CTCGT [b] Pre vs Post	8.15	8.110 to 8.207	< 0.001
	PTCGT [a] Pre vs CTCGT [b] Pre	1.16	−0.561 to 2.893	0.181
	PTCGT [a] Post vs CTCGT [b] Post	13.16	11.413 to 14.921	< 0.001
Paretic Step Length (cm)	PTCGT [a] Pre vs Post	10.36	10.013 to 10.717	< 0.001
	CTCGT [b] Pre vs Post	3.91	3.842 to 3.989	< 0.001
	PTCGT [a] Pre vs CTCGT [b] Pre	0.79	−0.370 to 1.950	0.178
	PTCGT [a] Post vs CTCGT [b] Post	7.23	6.230 to 8.247	< 0.001
Non-paretic Step Length (cm)	PTCGT [a] Pre vs Post	4.18	3.927 to 4.437	< 0.001
	CTCGT [b] Pre vs Post	1.62	1.558 to 1.692	< 0.001
	PTCGT [a] Pre vs CTCGT [b] Pre	0.68	−0.382 to 1.753	0.205
	PTCGT [a] Post vs CTCGT [b] Post	3.24	2.293 to 4.193	< 0.001
Paretic Swing (%)	PTCGT [a] Pre vs Post	5.73	5.604 to 5.872	< 0.001
	CTCGT [b] Pre vs Post	1.37	1.322 to 1.421	< 0.001
	PTCGT [a] Pre vs CTCGT [b] Pre	0.53	−0.110 to 1.185	0.102
	PTCGT [a] Post vs CTCGT [b] Post	4.90	4.319 to 5.490	< 0.001
Non-paretic Swing (%)	PTCGT [a] Pre vs Post	1.69	1.574 to 1.824	< 0.001
	CTCGT [b] Pre vs Post	0.77	0.759 to 0.793	< 0.001
	PTCGT [a] Pre vs CTCGT [b] Pre	0.55	0.008 to 1.095	0.047
	PTCGT [a] Post vs CTCGT [b] Post	1.47	1.006 to 1.944	< 0.001
Paretic Stance (%)	PTCGT [a] Pre vs Post	-5.78	−5.913 to −5.664	< 0.001
	CTCGT [b] Pre vs Post	-1.37	−1.421 to −1.321	< 0.001
	PTCGT [a] Pre vs CTCGT [b] Pre	-0.46	−1.103 to 0.168	0.145

	PTCGT [a] Post vs CTCGT [b] Post	-4.88	−5.468 to −4.302	< 0.001
Non-paretic Stance (%)	PTCGT [a] Pre vs Post	-1.69	−1.824 to −1.574	< 0.001
	CTCGT [b] Pre vs Post	-0.77	−0.790 to −0.758	< 0.001
	PTCGT [a] Pre vs CTCGT [b] Pre	-0.56	−1.110 to −0.026	0.040
	PTCGT [a] Post vs CTCGT [b] Post	-1.49	−1.961 to −1.025	< 0.001
Paretic Single Support cycle (%)	PTCGT [a] Pre vs Post	6.32	6.143 to 6.505	< 0.001
	CTCGT [b] Pre vs Post	1.33	1.301 to 1.369	< 0.001
	PTCGT [a] Pre vs CTCGT [b] Pre	0.45	−0.205 to 1.116	0.175
	PTCGT [a] Post vs CTCGT [b] Post	5.44	4.734 to 6.155	< 0.001
Non-paretic Single Support cycle (%)	PTCGT [a] Pre vs Post	3.89	3.758 to 4.027	< 0.001
	CTCGT [b] Pre vs Post	1.25	1.233 to 1.275	< 0.001
	PTCGT [a] Pre vs CTCGT [b] Pre	0.51	−0.133 to 1.159	0.118
	PTCGT [a] Post vs CTCGT [b] Post	3.15	2.482 to 3.822	< 0.001
Double Support cycle (%)	PTCGT [a] Pre vs Post	-9.21	−9.446 to −8.979	< 0.001
	CTCGT [b] Pre vs Post	-3.61	−3.765 to −3.455	< 0.001
	PTCGT [a] Pre vs CTCGT [b] Pre	-0.57	−1.360 to 0.217	0.153
	PTCGT [a] Post vs CTCGT [b] Post	-6.17	−6.813 to −5.536	< 0.001

a. PTCGT: Progressive Task-Complexity Gait Training group; b. CTCGT: Constant Task-Complexity Gait Training group; c. CI: Confidence Interval. The mean differences and their 95% CIs were computed in SPSS for the pairwise comparisons specified in the Comparison column, including within-group pre–post and between-group pre- or post-intervention comparisons.

Table S6. Changes in electroencephalography (EEG) power spectrum variables before and after intervention (N = 44)

Variable		Group	Pre (Mean ± SD [c])	Post (Mean ± SD [c])	Time (F, p)	ηp² [d]	Group (F, p)	ηp² [d]
Theta	Rest	PTCGT [a]	19.22 ± 1.37	18.49 ± 1.18	F=213.822, p < 0.001	0.836	F=1.059, p = 0.309	0.025
		CTCGT [b]	19.28 ± 1.43	19.28 ± 1.37				
	Single	PTCGT [a]	17.65 ± 1.24	16.42 ± 0.84	F=357.488, p < 0.001	0.895	F=2.828, p = 0.100	0.063
		CTCGT [b]	17.69 ± 1.11	17.53 ± 1.06				
	Dual	PTCGT [a]	20.48 ± 1.82	18.10 ± 1.13	F=355.043, p < 0.001	0.894	F=5.741, p = 0.021	0.120
		CTCGT [b]	20.55 ± 1.74	20.25 ± 1.61				
SMR	Rest	PTCGT [a]	8.30 ± 0.67	8.79 ± 0.80	F=285.628, p < 0.001[†]	0.872	F=0.625, p = 0.434	0.015
		CTCGT [b]	8.31 ± 0.68	8.39 ± 0.69				
	Single	PTCGT [a]	9.50 ± 0.79	10.75 ± 1.06	F=338.329, p < 0.001[†]	0.890	F=4.957, p = 0.031	0.106
		CTCGT [b]	9.50 ± 0.78	9.61 ± 0.80				
	Dual	PTCGT [a]	10.30 ± 0.96	12.08 ± 1.28	F=356.042, p < 0.001[†]	0.894	F=7.084, p = 0.011	0.144
		CTCGT [b]	10.32 ± 0.97	10.43 ± 1.00				
Mid-Beta	Rest	PTCGT [a]	7.47 ± 0.66	8.00 ± 0.81	F=233.150, p < 0.001[†]	0.847	F=0.908, p = 0.346	0.021
		CTCGT [b]	7.46 ± 0.67	7.54 ± 0.69				
	Single	PTCGT [a]	8.42 ± 0.86	9.85 ± 1.18	F=342.845, p < 0.001[†]	0.891	F=6.354, p = 0.016	0.131
		CTCGT [b]	8.37 ± 0.82	8.47 ± 0.85				
	Dual	PTCGT [a]	9.46 ± 1.06	11.50 ± 1.39	F=393.917, p < 0.001[†]	0.904	F=8.657, p = 0.005	0.171
		CTCGT [b]	9.44 ± 1.06	9.52 ± 1.07				

a. PTCGT: Progressive Task-Complexity Gait Training group; b. CTCGT: Constant Task-Complexity Gait Training group; c. SD: Standard Deviation; d. ηp²: partial eta squared. [†] $p < .01$, Bonferroni-adjusted significance threshold for the five primary outcomes.

Table S7. Changes in Spectral Edge Frequency 50% (SEF-50) before and after intervention (*N* = 44)

Variable	Group	Pre (Mean ± SD [c])	Post (Mean ± SD [c])	Time (F, p)	ηp² [d]	Group (F, p)	ηp² [d]
Rest	PTCGT [a]	9.57 ± 0.35	9.86 ± 0.42	F=301.938, $p < 0.001^{\dagger}$	0.878	F=0.687, p = 0.412	0.016
	CTCGT [b]	9.57 ± 0.35	9.66 ± 0.39				
Single	PTCGT [a]	10.15 ± 0.41	10.89 ± 0.51	F=489.531, $p < 0.001^{\dagger}$	0.921	F=5.801, p = 0.02	0.121
	CTCGT [b]	10.17 ± 0.39	10.25 ± 0.41				
Dual	PTCGT [a]	10.84 ± 0.47	11.60 ± 0.54	F=325.211, $p < 0.001^{\dagger}$	0.886	F=5.276, p = 0.027	0.112
	CTCGT [b]	10.85 ± 0.49	10.93 ± 0.50				

a. PTCGT: Progressive Task-Complexity Gait Training group; b. CTCGT: Constant Task-Complexity Gait Training group; c. SD: Standard Deviation; d. ηp²: partial eta squared. † p < .01, Bonferroni-adjusted significance threshold for the five primary outcomes.

Table S8. Changes in Concentration Index (CI) before and after intervention (*N* = 44)

Variable	Group	Pre (Mean ± SD [c])	Post (Mean ± SD [c])	Time (F, p)	ηp² [d]	Group (F, p)	ηp² [d]
Rest	PTCGT [a]	0.81 ± 0.03	0.94 ± 0.08	F=176.946, p < 0.001	0.808	F=35.188, p < 0.001	0.456
	CTCGT [b]	0.83 ± 0.04	0.88 ± 0.22				
Single	PTCGT [a]	1.04 ± 0.08	1.25 ± 0.07	F=99.110, p < 0.001	0.702	F=66.452, p < 0.001	0.613
	CTCGT [b]	1.03 ± 0.13	1.04 ± 0.08				
Dual	PTCGT [a]	0.97 ± 0.08	1.30 ± 0.08	F=114.692, p < 0.001	0.732	F=88.820, p < 0.001	0.679
	CTCGT [b]	0.94 ± 0.09	0.99 ± 0.07				

a. PTCGT: Progressive Task-Complexity Gait Training group; b. CTCGT: Constant Task-Complexity Gait Training group; c. SD: Standard Deviation; d. ηp²: partial eta squared.

Table S9. Changes in Dual-Task Cost (DTC) before and after intervention (*N* = 44)

Variable	Group	Pre (Mean ± SD [c])	Post (Mean ± SD [c])	Time (F, p)	ηp² [d]	Group (F, p)	ηp² [d]
DTC [e] (%)	PTCGT [a]	14.17 ± 5.21	8.75 ± 3.46	F=13.594, p < 0.001	0.245	F=16.490, p < 0.001	0.282
	CTCGT [b]	15.41 ± 4.12	13.26 ± 3.41				

a. PTCGT: Progressive Task-Complexity Gait Training group; b. CTCGT: Constant Task-Complexity Gait Training group; c. SD: Standard Deviation; d. ηp²: partial eta squared; e. DTC: Dual-task cost

Table S10. Changes in Spatiotemporal Gait Variables Assessed Using GAITRite before and after intervention (*N* = 44)

Variable	Group	Pre (Mean ± SD [c])	Post (Mean ± SD [c])	Time (F, p)	ηp² [d]	Group (F, p)	ηp² [d]
Velocity (cm/s)	PTCGT [a]	56.24 ± 2.55	72.30 ± 2.70	F=5579.896, p < 0.001	0.993	F=67.904, p < 0.001	0.618
	CTCGT [b]	55.41 ± 1.44	63.47 ± 1.39				
Cadence (steps/min)	PTCGT [a]	85.45 ± 3.01	97.20 ± 2.80	F=4920.824, p < 0.001	0.992	F=43.754, p < 0.001	0.510
	CTCGT [b]	84.47 ± 1.74	89.38 ± 1.52				
Stride length (cm)	PTCGT [a]	74.15 ± 3.45	94.31 ± 3.48	F=7254.379, p < 0.001	0.994	F=97.442, p < 0.001	0.699
	CTCGT [b]	72.99 ± 1.99	81.14 ± 2.07				
Paretic Step Length (cm)	PTCGT [a]	37.55 ± 2.36	47.91 ± 2.04	F=6056.480, p < 0.001	0.993	F=74.354, p < 0.001	0.639
	CTCGT [b]	36.76 ± 1.23	40.68 ± 1.10				
Non-paretic Step Length (cm)	PTCGT [a]	45.62 ± 2.10	49.80 ± 1.86	F=2096.928, p < 0.001	0.980	F=15.837, p < 0.001	0.274
	CTCGT [b]	44.93 ± 1.28	46.56 ± 1.16				
Paretic Swing (%)	PTCGT [a]	32.31 ± 1.31	38.04 ± 1.18	F=8492.146, p < 0.001	0.995	F=114.124, p < 0.001	0.731
	CTCGT [b]	31.77 ± 0.71	33.14 ± 0.66				
Non-paretic Swing (%)	PTCGT [a]	38.67 ± 1.12	40.37 ± 0.94	F=616.297, p < 0.001	0.936	F=17.007, p < 0.001	0.288
	CTCGT [b]	38.12 ± 0.55	38.90 ± 0.54				
Paretic Stance (%)	PTCGT [a]	67.76 ± 1.28	61.98 ± 1.17	F=9416.820, p < 0.001	0.996	F=113.906, p < 0.001	0.731
	CTCGT [b]	68.23 ± 0.71	66.86 ± 0.66				
Non-paretic Stance (%)	PTCGT [a]	61.33 ± 1.12	59.63 ± 0.94	F=609.907, p < 0.001	0.936	F=18.327, p < 0.001	0.304
	CTCGT [b]	61.90 ± 0.54	61.12 ± 0.54				
Paretic Single Support cycle (%)	PTCGT [a]	26.18 ± 1.31	32.51 ± 1.46	F=7486.058, p < 0.001	0.994	F=77.995, p < 0.001	0.650
	CTCGT [b]	25.73 ± 0.78	27.06 ± 0.73				
Non-paretic Single Support cycle (%)	PTCGT [a]	31.87 ± 1.31	35.76 ± 1.36	F=2902.098, p < 0.001	0.986	F=36.704, p < 0.001	0.466
	CTCGT [b]	31.36 ± 0.70	32.61 ± 0.72				
Double Support cycle (%)	PTCGT [a]	29.85 ± 1.56	20.63 ± 1.33	F=9039.961, p < 0.001	0.995	F=95.123, p < 0.001	0.694
	CTCGT [b]	30.42 ± 0.94	26.81 ± 0.61				

a. PTCGT: Progressive Task-Complexity Gait Training group; b. CTCGT: Constant Task-Complexity Gait Training group; c. SD: Standard Deviation; d. ηp²: partial eta squared

Table S11. Sensitivity Analysis Using Linear Mixed-Effects Models for Group × Time Interactions in EEG-Derived Outcomes

Outcome	Variable	Estimate	95% CI [a]	t	*p*
Theta	Rest	0.73	0.63 to 0.82	15.223	< 0.001
	Single	1.07	0.89 to 1.25	11.858	< 0.001
	Dual	2.08	1.77 to 2.39	13.487	< 0.001
SMR	Rest	-0.41	-0.47 to -0.35	-13.369	< 0.001
	Single	-1.12	-1.25 to -1.00	-18.481	< 0.001
	Dual	-1.67	-1.81 to -1.52	-22.979	< 0.001
Mid-Beta	Rest	-0.45	-0.53 to -0.37	-11.883	< 0.001
	Single	-1.33	-1.48 to -1.18	-18.033	< 0.001
	Dual	-1.96	-2.11 to -1.71	-25.652	< 0.001
CI	Rest	-0.08	-0.09 to -0.05	-11.065	< 0.001
	Single	-0.21	-0.23 to -0.19	-19.217	< 0.001
	Dual	-0.31	-0.33 to -0.28	-25.996	< 0.001
SEF	Rest	-0.20	-0.25 to -0.15	-8.558	< 0.001
	Single	-0.65	-0.71 to -0.60	-23.899	< 0.001
	Dual	-0.69	-0.73 to -0.64	-32.691	< 0.001

a. CI: Confidence Interval. The estimates and their 95% CIs were obtained for the Group × Time interactions from the linear mixed-effects models using SPSS.

Table S12. Sensitivity Analysis Using a Linear Mixed-Effects Model for the Group × Time Interaction in Dual-Task Cost (DTC)

Variable	Estimate	95% CI [a]	t	p
DTC [b] (%)	-0.23	-0.51 to 0.03	-1.747	0.088

a. CI: Confidence Interval; b. DTC: Dual-Task Cost. The estimates and their 95% CIs were obtained for the Group × Time interactions from the linear mixed-effects models using SPSS.

Table S13 Sensitivity Analysis Using Linear Mixed-Effects Models for Group × Time Interactions in Spatiotemporal Gait Variables

Variable	Estimate	95% CI [a]	t	*p*
Velocity (cm/s)	-8.01	-8.43 to -7.58	-38.192	< 0.001
Cadence (steps/min)	-6.84	-7.31 to -6.37	-29.369	< 0.001
Stride length (cm)	-12.00	-12.46 to -11.53	-52.193	< 0.001
Paretic Step Length (cm)	-6.44	-6.79 to -6.10	-37.34	< 0.001
Non-paretic Step Length (cm)	-2.55	-2.81 to -2.30	-20.163	< 0.001
Paretic Swing (%)	-4.36	-4.50 to -4.22	-63.631	< 0.001
Non-paretic Swing (%)	-0.92	-1.63 to -0.22	-2.619	< 0.001
Paretic Stance (%)	4.41	3.57 to 5.26	10.422	< 0.001
Non-paretic Stance (%)	0.92	0.22 to 1.62	2.631	< 0.001
Paretic Single Support cycle (%)	-4.98	-5.93 to -4.04	-10.470	< 0.001
Non-paretic Single Support cycle (%)	-2.63	-3.54 to -1.72	-5.775	< 0.001
Double Support cycle (%)	5.60	4.61 to 6.59	11.243	< 0.001

a. CI: Confidence interval. The estimates and their 95% CIs were obtained for the Group × Time interactions from the linear mixed-effects models using SPSS.
